# [Retracted] Electroacupuncture attenuates cervical spinal cord injury following cerebral ischemia/reperfusion in stroke‑prone renovascular hypertensive rats

**DOI:** 10.3892/etm.2024.12726

**Published:** 2024-09-24

**Authors:** Feng Tan, Jie Chen, Yangui Liang, Minhua Gu, Yanping Li, Xuewen Wang, Di Meng

Exp Ther Med 7:1529–1534, 2014; DOI: 10.3892/etm.2014.1619

Following the publication of this article, a concerned reader drew to the Editor’s attention that, with the immunohistochemical experiments shown in Fig. 1 on p. 1531, multiple and unexpected areas of similarity were identified in terms of cellular patterns and motifs comparing both within and between the data panels A-L.

After having conducted an internal investigation, the Editor of *Experimental and Therapeutic Medicine* has reached the conclusion that the overlapping sections of data shown in this figure were unlikely to have arisen by coincidence. Therefore, on the grounds of a lack of confidence in the integrity of these data, the Editor has decided that the article should be retracted from the publication. The authors were asked for an explanation to account for these concerns, but the Editorial Office did not receive a reply. The Editor apologizes to the readership for any inconvenience caused, and thanks the interested reader for drawing this matter to our attention.

